# The Contribution of Qualitative CEUS to the Determination of Malignancy in Adnexal Masses, Indeterminate on Conventional US – A Multicenter Study

**DOI:** 10.1371/journal.pone.0093843

**Published:** 2014-04-15

**Authors:** Xinling Zhang, Yongjiang Mao, Rongqin Zheng, Zhijuan Zheng, Zeping Huang, Dongmei Huang, Jing Zhang, Qing Dai, Xiaodong Zhou, Yanling Wen

**Affiliations:** 1 Department of Ultrasound, Third Affiliated Hospital of Sun Yat-sen University, Guangzhou, China; 2 Department of Ultrasound, General Hospital of Chinese People’s Liberation Army, Beijing, China; 3 Department of Ultrasound, Peking Union Medical College Hospital, Beijing, China; 4 Department of Ultrasound, Xi Jing Hospital of Fourth Military Medical University, Xi’an, China; 5 Department of Ultrasound, Sixth Affiliated Hospital of Sun Yat-sen University, Guangzhou, China; Institute of Automation, Chinese Academy of Sciences, China

## Abstract

The aim of this study is to evaluate the efficacy of qualitative analysis of contrast-enhanced ultrasound (CEUS) in discrimination of adnexal masses which were undetermined by conventional ultrasound (US). A total of 120 patients underwent transabdominal CEUS. The initial enhancement time and intensity compared with the uterine myometrium, contrast agent distribution patterns and dynamic changes of enhancement were assessed. The sensitivity (Sen), specificity (Spe), positive predictive value (PPV), negative predictive value (NPV), accuracy (ACC) and Youden’s index were calculated for contrast variables. The gold standard was the histological diagnosis. There were 48 malignant tumors and 72 benign tumors. The enhancement features of malignant masses were different from benign ones. Earlier or simultaneous enhancement with inhomogeneous enhancement yielded the highest capability in differential diagnosis, and Sen, Spe, PPV, NPV, ACC, Youden’s index was 89.6%, 97.2%, 93.2%, 95.6%, 93.3%, and 0.88, respectively. The qualitative evaluation of CEUS is useful in the differential diagnosis of adnexal masses where conventional US is indeterminate.

## Introduction

An adnexal mass is not a common disease. It is a finding or an observation of physical examination or imaging. To determine the nature of the masses is essential for appropriate management. Ultrasound (US) is considered the primary imaging modality for detection of the adnexal mass. However, it has difficulty in characterization of some cases, especially in differentiating extra-uterine myomas with cystic degeneration from ovarian carcinoma and corpus luteum with solid component from malignant masses [1]–[4]. Color, power and spectral Doppler US are widely used in discrimination of malignant from benign adnexal diseases. However, there is no consensus as to which Doppler parameters and cutoff values are the most predictive of malignancy because of the overlap in blood flow parameters between benign and malignant lesions. In addition, conventional Doppler US has inherent limitations, such as inferior sensitivity to slow flow and deeply located blood vessels [5]–[7]. 3-D power Doppler US provides a new method to evaluate the tumor vascularity. Combined evaluations of morphology and neovascularization by 3-D power Doppler US may improve the detection of ovarian carcinoma [8]–[9]. But it is controversial about the descriptive and qualitative 3-D power Doppler criteria to evaluate the ovarian tumor microcirculation. It has also been reported that 3-D power Doppler imaging does not improve diagnostic performance compared with 2-D power Doppler imaging in characterization of complex adnexal masses [10]–[11].

Real-time contrast-enhanced ultrasound (CEUS) technology, e.g., cadence contrast pulse sequencing (CPS) imaging mode with low mechanical index (MI) technique using a second generation contrast agent Sonovue (sulfur hexafluoride microbubbles, Bracco Imaging S.p.a., Milan, Italy), overcomes the limitations of conventional US and greatly improves the ability to depict vascularity in tumors [12]–[14]. With low-MI (lower than 0.3) imaging technique, the UCA would not be destroyed and could remain in the blood for several minutes. Thus, the dynamic perfusion of the UCA in the tumor can be continuously observed and the tissue signals from background can be effectively suppressed. Currently, it is widely used in the discrimination of liver tumors [15]–[17], while there are few reports about low-MI CEUS in the differential diagnosis of adnexal masses [18]–[21]. Some of the enhancement features are co-existence in benign, borderline and malignant masses, which make it difficult to discriminate the adnexal masses. The aim of this multicenter study is to determine whether the qualitative parameters of CEUS are useful in characterization of adnexal masses which are indeterminate by conventional US.

## Methods

From December 2007 to April 2010, 184 consecutive patients who had indeterminate adnexal tumors by routing conventional US were enrolled for CEUS studies in5 ultrasound centers under the same protocol. The study was approved by ethics committees of the participating institutes, including Sixth Affiliated Hospital of Sun-Yat Sen University, Third Affiliated Hospital of Sun-Yat Sen University; General Hospital of Chinese People’s Liberation Army, Peking Union Medical College Hospital; and Xi Jing Hospital of Fourth Military Medical University.

The inclusion criteria were: (1)ultrasound diagnosis of unilocular-solid(a single cyst containing solid parts or papillary excrescences but no septa), multilocular-solid (a cyst with at least one septum and solid parts or papillary excrescences) or solid adnexal mass (a tumor with solid components in 80% or more of the tumor) or multilocular adnexal cyst (a cyst with more than one septum but no solid parts or papillary excrescences), (2) negative pregnancy test, (3)surgery planed with pathology results within 3 months, and (4) the mass and the myometrium seen in the same imaging plane. The written informed consent was obtained from each patient after full explanation of the nature and process of the procedure to the candidate. Patients who had known or suspected cardiovascular and pulmonary diseases such as cardiac insufficiency, coronary heart disease, and pulmonary hypertension, those who were critically ill, and who were older than 80 years or younger than 18 years were excluded from the study. Path of borderline tumors were classified as malignant in order to analysis.

Among 180 patients, nineteen were excluded because the mass and myometrium could not be showed in the same imaging plane. Thirty seven patients were excluded because they did not undergo surgery within 3 months after CEUS study. The other eight patients failed to be followed up. Thus, a total of 120 patients were included and analyzed in the study eventually. The patients’ number of Second Affiliated Hospital of Sun-Yat Sen University, Third Affiliated Hospital of Sun-Yat Sen University; General Hospital of Chinese People’s Liberation Army, Peking Union Medical College Hospital; and Xi Jing Hospital of Fourth Military Medical University is 18,50,32,17 and 13 respectively. There were 48 malignant tumors and 72 benign tumors which were confirmed by surgical pathology. (Table 1). No adverse effects of SonoVue and technical related problems occurred in this study. The patients’ demography, conventional gray-scale and Doppler US findings are showed in Table 2. The mean age of patients with malignant masses was older than those with benign lesions with statistically significant difference (*p* = 0.001). Significant difference was also found between two groups in term of the ascites (*p*<0.001), while there were no statistically significant differences between the two groups for size and location of the masses (*p*>0.05). The highest Doppler score of lesion vascularity was 47.9% (23/48) in malignant masses while only 1.4% (1/72) in benign lesions (*p*<0.001).

**Table 1:** Pathological types of 120 pelvic masses.

benign	n	malignant		n
Endometrial cyst	22	Cystadenocarcinoma		27
Cystadenoma	21	Metastatic tumor		4
Mature teratoma	14	Immature teratoma		4
orpus luteum	10	Cleare cell carcinoma		2
Pelvic abscess	4	Endometrioid adenocarcinoma		2
Cyst torsion	1	Squamous cells carcinoma		1
		Endodermal sinus tumor		1
		Borderline cystadenoma		7
Total	72			48

**Table 2:** Demography and US findings of 120 adnexal masses.

parameter	malignant	benign	P**
Age*	42±16	38±13	0.001
Size (cm)*	10.05±4.68	7.57±3.87	0.070
bilateral	13(27.1%)	10(13.9%)	0.072
unilateral	35(72.9%)	62(86.1%)	
Ascites	28(58.3%)	14(19.4%)	0.000
vascularity			
1	0	17(23.6%)	0
2	15(31.3%)	44(61.1%)	0.001
3	10(20.8%)	10(13.9%)	0.317
4	23(47.9%)	1(1.4%)	0.000

*Data are expressed as . **Independent *t*-tests for continuous variables and Chi-square tests or Fisher’s exact test for other variables.

Conventional US and CEUS examinations were performed by US doctors who had more than 5-years’ experience in gynecological CEUS and were not involved in later evaluation of US and CEUS images. Conventional US examination was carried out first and then CEUS was followed. Two types of US machines were used in the study. One was Volusion730 Expert (GE medical system, Zips, Austria) equipped with a high-resolution endovaginal probe (RIC 5-9H), which mainly was used to perform conventional US examination. The other was Acuson Sequoia 512 system (Siemens Medical Solutions, Mountain View, Calif.) equipped with CPS. A 4V1 vector transducer with a frequency range of 1–4 MHz was used mainly for CEUS examination. A transvaginal gray-scale and color/power Doppler US examination was performed using a standardized examination technique and standardized color/power Doppler settings (frequency 7.5 MHz, pulse repetition frequency 900 Hz, color gain just below the background noise level). The location, size, shape, internal echogenicity of the mass, ascites and vascularity were recorded. In the patients with multiple masses, the largest or most difficult-to-diagnosis mass was selected for evaluation. A color Doppler score system that was described by Timmerman et al [22]. (1 = no vascularization; 2 = minimal vascularization; 3 = moderate vascularization, 4 = high vascularization) was used to evaluate the vascularity of the masses. The CPS function was activated after contrast injection with contrast-GYN mode and 0.15–0.21 low MI setting. The same parameters were used for all contrast examinations including gain 21–26 dB, dynamic range 76 dB, space/time S1, edge 0, persistent 3, postprocess 4, and delta 4. Each patient received SonoVue at a dose of 2.4-mL by intravenous bolus injection via an indwelling catheter placed in antecubital vein (20-gauge Venflon; Becton Dickinson, Helsingborg, Sweden). The agent’s diameter is less than 8 µm (mean 2.5 µm), the concentration is 8 µl/ml. The target lesion was observed continuously for 3 minutes after the contrast agent injection. The entire imaging files were recorded and saved on the built-in hard disk. All the data were completely integrated for further analysis.

Digital cine clips of CEUS studies were analyzed off-line. Images were analyzed in consensus by two experienced investigators, who were neither involved in the US and CEUS examinations nor aware of the clinical history and the results of other imaging and pathological findings.

For CEUS analysis, the initial enhancement time of the mass was determined as earlier than, simultaneous with and later than the enhancement of the myometrium. The level of the enhancement of the lesion was compared to the adjacent myometrium and described as hypo, iso or hyper enhancing. If variable intensities were seen within the lesion, the highest degree of enhancement was selected for the classification. The distribution patterns of enhancement were divided into homogeneous and heterogeneous enhancement based on the uniform or non-uniform of distribution and intensity of contrast agent within the lesion. During the washout phase, the dynamic change of the enhancement as compared with the uterine myometrium from hyper- or iso-enhancement to hypo-enhancement was observed and recorded.

The value of each enhancement feature and the various combinations of these features in differentiating diagnosis between benign and malignant adnexal diseases was calculated. The sensitivity (Sen), specificity (Spe), positive predictive value (PPV), negative predictive value (NPV), accuracy (ACC), and Youden’s index were calculated for contrast variables.

### Statistical Analysis

Continuous data are expressed as the mean±standard deviation. Independent *t*-tests were applied to evaluate the difference between benign and malignant adnexal masses in terms of patients’ age and the size of lesion. Chi-square tests were applied to evaluate the difference between benign and malignant masses in the echogenicity, intralesional vascularity on conventional US, and enhancement time, intensity, the agent distribution patterns and the dynamic changes on CEUS. A two-tailed p value of less than 0.05 was considered statistically significant. The Youden’s index was calculated as Sen+Spe-1. The statistical analysis was performed with the SPSS 17.0 software package (SPSS Inc., Chicago, IL).

## Results

**Figure 1:**
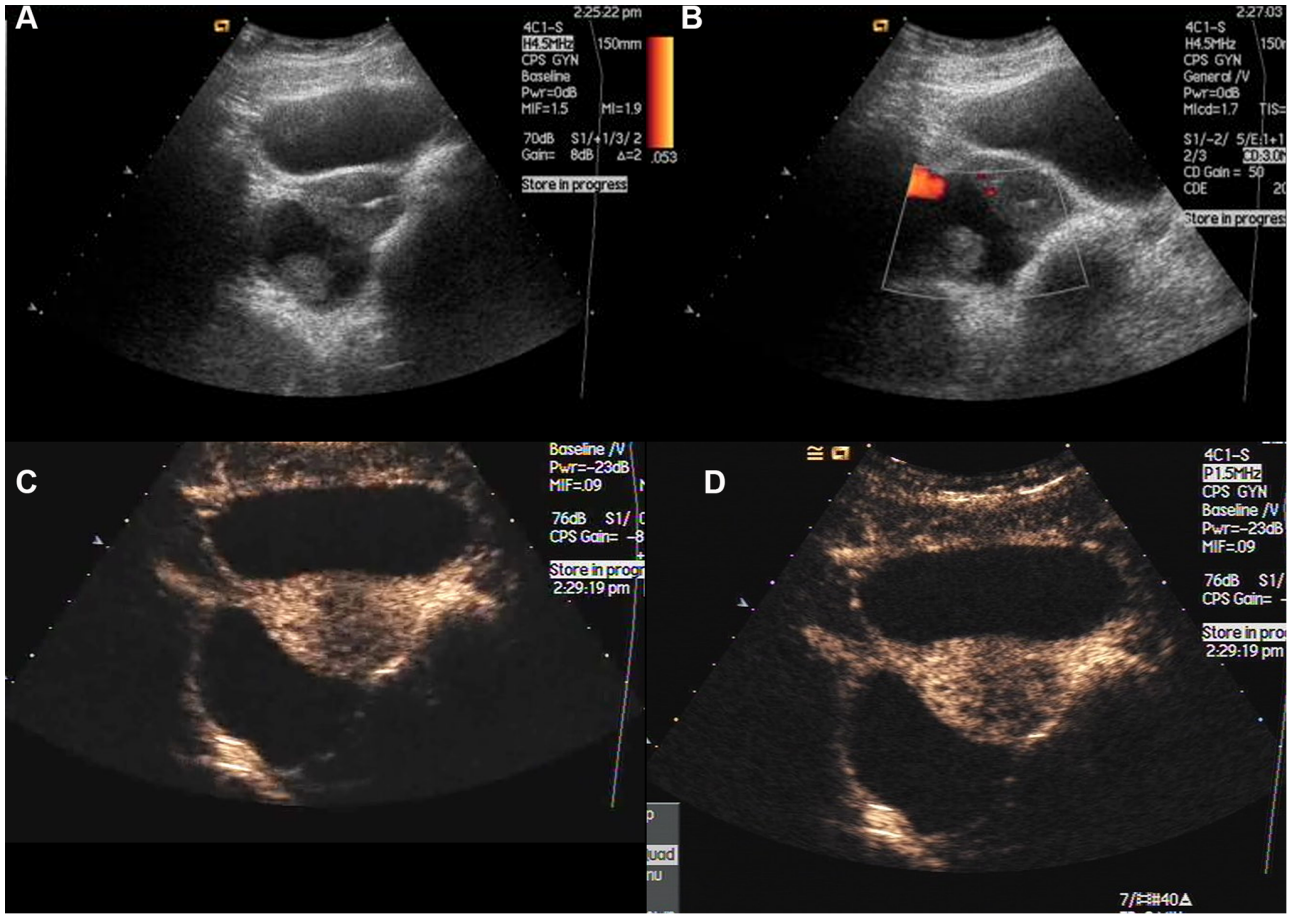
A 21-year-old woman with endometrial cyst. A: Gray-scale sonogram shows a 6.2-cm inhomogeneous mass with solid projection at the right of uterus. B: Power Doppler shows minimal vascularization. C: CEUS scan at 9 s shows later enhancement in comparison with myometrium (arrow), no agent perfusion inside. D: CEUS scan at 17 s shows homogeneous ringed (arrow).

**Figure 2:**
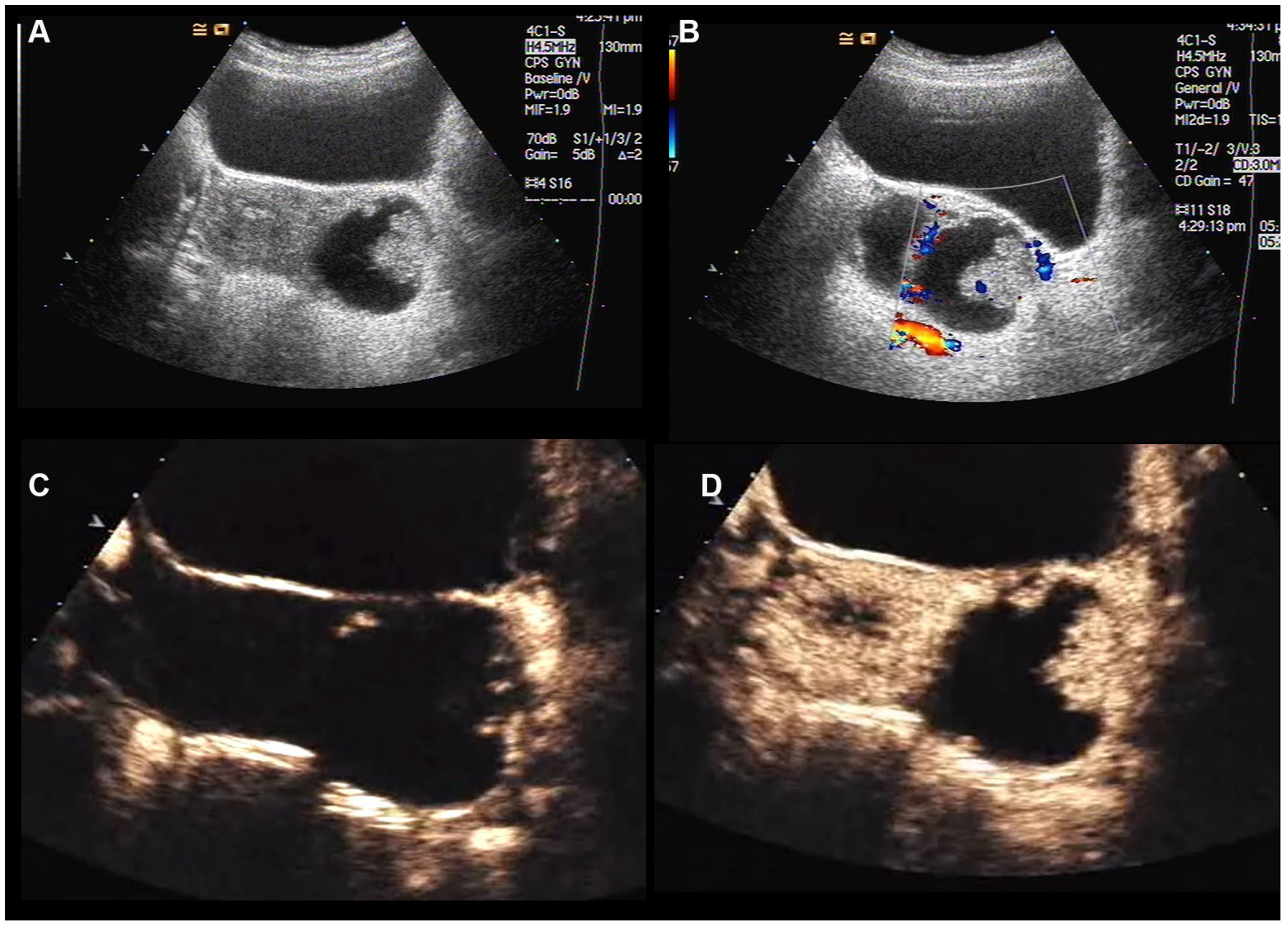
A 22-year-old woman with serous cystadenocarcinoma. A: Gray-scale sonogram shows an 5.2-cm echogenic mass (arrow) with solid projection at the left of uterus. B: Color Doppler shows minimal-moderate vascularization. C: CEUS scan at 12 s shows earlier enhancement (arrow) in comparison with myometrium. D: CEUS scan at 15 s shows inhomogeneous hyper-enhancement with agent perfusion inside (arrow).

The initial enhancement time compared with the myometrium is shown in Table 3. Earlier, simultaneous and later enhancements (As shown in Fig. 1c) were found in 58.3% (28/48), 35.4% (17/48) and 6.3% (3/48) of malignant adnexal masses, and 4.2% (3/72), 8.3% (6/72) and 87.5% (63/72) of benign lesions, respectively. Earlier and simultaneous enhancements were more commonly seen in malignant masses (As shown in Fig. 2c) (93.8% 45/48) than in benign lesions (12.5% 9/72) (*p*<0.001).

**Table 3:** The initial enhancement time of adnexal masses compared with myometrium on CEUS.

Enhancement time	malignant	benign	p
Earlier (E)	28(58.3%)	3(4.2%)	0.000
Simultaneous (S)	17 (35.4%)	6 (8.3%)	0.000
Later (L)	3 (6.3%)	63 (87.5%)	0.000
E+S	45(93.8%)	9(12.5%)	0.000

The enhancement level is shown in Table 4. Hyper-enhancement was more common in malignant masses (79.2% 38/48) than in benign lesions (15.3% 11/72) while iso-enhancement was more often seen in benign lesions (69.4% 50/72) than in the malignant (18.8% 9/48) with significant difference between the two groups (*p*<0.05). No mass showed non-enhancement in either of the categories.

**Table 4:** The enhancement degree of the adnexal masses on CEUS.

Enhancement extent	malignant	benign	p
Hyper-	38 (79.2%)	11(15.3%)	0.000
Iso-	9 (18.8%)	50(69.4%)	0.000
Hypo-	1 (2.1%)	11(15.3%)	0.040
Hyper+Iso	47(97.9%)	61(84.7%)	0.040

The contrast agent distribution patterns of adnexal masses are listed in Table 5. The malignant lesions were characterized by heterogeneous enhancement (As shown in Fig. 2d) (91.7% (44/48) VS 6.9% (5/72) *p*<0.001).Whereas, the homogeneous enhancement was common in benign lesions (As shown in Fig. 1d) (93.1%, 67/72).

**Table 5:** The contrast agent distribution patterns of the adnexal masses on CEUS.

Distribution	Malignant	Benign	p
Homogeneous	4(8.3%)	67(93.1%)	
Heterogeneous	44(91.7%)	5(6.9%)	0.000

The dynamic changes of enhancement are shown in Table 6. During the wash-out phase, i.e., the hyper-enhancement or iso-enhancement fading out to hypo-enhancement happened in 42 (87.5% 42/48) adnexal carcinoma and 57 (79.2% 57/72) benign adnexal diseases (*p*>0.05).

**Table 6:** The dynamic changes of enhancement of the adnexal masses on CEUS.

Dynamic changes of enhancement	malignant	benign	p
hyper- to hypo-	33(68.8%)	8(11.1%)	0.000
hyper- to iso-	3(6.3%)	3(4.2%)	0.932
iso- to hypo-	9(18.8%)	49(68.1%)	0.000
remained hyper-	2(4.2%)		
remained hypo-	1(1.9%)	11(15.3%)	
iso- to iso-		1(1.3%)	
hyper−/iso- to hypo-	42(87.5%)	57(79.2%)	0.239

The earlier or simultaneous enhancement, hyper- or iso-enhancement and heterogeneous enhancement were found in 45 (93.8% 45/48), 47 (97.9% 47/48), and 43 (87.6% 43/48) of 48 adnexal carcinomas, respectively. The diagnostic performance of these features in differentiating malignant and benign adnexal masses, either individual or the combination of each two or three, were calculated and are listed in Table 7. Diagnostic test found that earlier or simultaneous enhancement combine heterogeneous enhancement yielded the highest diagnostic capability in the discrimination of adnexal masses, with the sensitivity of 89.6% (43/48), specificity of 97.2% (70/72), positive predictive value of 93.2% (43/45), negative predictive value of 95.6% (70/75), ACC of 93.3% (113/120), Youden’s index of 0.88, respectively.

**Table 7:** The diagnostic capability of CEUS in differential diagnosis of adnexal masses.

	Sensitivity	Specificity	PPV	NPV	Accuracy	Youden’s index
A	93.8	87.5	83.3	95.5	90	0.81
B	97.9	15.3	43.5	91.7	48.3	0.13
C	91.7	88.9	84.6	94.1	90	0.81
A+B	93.8	88.9	84.9	95.5	90.8	0.83
A+C	89.6	97.2	93.2	95.6	93.3	0.88
B+C	85.4	90.3	85.4	90.3	90	0.76
A+B+C	83.3	97.2	95.2	89.7	91.2	0.81

A Earlier or simultaneous beginning of enhancement; B Hyper- or iso-enhancment; C Heterogeneous enhancement.

Among the 48 cases with malignant adnexal tumor, 41 (85.4% 41/48) were correctly diagnosed by CEUS. Two patients with pelvic abscess were misdiagnosed as carcinoma, which showed earlier hetero-hyper-enhancement. Five cases were improperly defined as benign disease, in which two of them were borderline cystadenoma with the feature of later homogenous enhancement and the other was cystadenocarcinoma with homogenous enhancement. Another one was an immature teratoma, which had later enhancement compared with myometrium. The last one was metastatic tumor from lung cancer, which remained hypo-enhancement throughout the wash-out phase.

## Discussion

Conventional US is a preferred imaging technique for diagnosis of adnexal masses. With the wide use of transvaginal color Doppler US, the diagnostic accuracy of malignant tumors has been improved dramatically. Whereas, gray-scale and Color Doppler US has been used to a certain extent to determine the nature of the adnexal cystic-solid masses, multilocular cyst and solid masses, especially in differentiating ovarian malignant tumors with some benign masses. The difficulties in differential diagnosis by conventional US (both gray-scale and Doppler imaging) are largely due to its low ability in depicting vascularity in adnexal masses, as neither color Doppler nor power Doppler is sensitive to slow flow and deeply located blood vessels [5], [23]–[24.

The real-time CEUS can overcome the limitations of conventional color or power Doppler US and improve the reflectivity of blood flow. Therefore it can reveal the microvascular component of the masses. Assessment of adnexal tumor neovascularity and discrimination of nature using low-MI CEUS are not common now, despite the wide use of color Doppler imaging in the differential diagnosis of liver, kidney, breast, pancreas and blunt abdominal trauma [16]–[17], [25]–[26]. To our knowledge, there were few studies on the discrimination of adnexal masses by CEUS with time intensity curves method. The contrast enhancement variables such as peak intensity, area under the intensity curve (AUC), time to peak (TTP), sharpness and half wash-out time were studied by Testa et al. and Fleischer et al. [19], [21], in which a second-generation contrast agent and low-MI CEUS were used. They found that AUC and peak values were highest in the malignant tumors and TTP values were similar in the benign and malignant tumors while a study by Sconfienza et al [20]. They found that TTP performed better than the other parameters. In other two studies led by Orden’ et al. and Marret et al. [27]–[28] with the use of a first-generation contrast agent (Levovist, Schering, Berlin, Germany) and standard power Doppler US for evaluation of adnexal masses, both of the studies showed that AUC value was the highest in the invasively malignant tumors, However, the TTP value had inconsistent findings in two studies. In addition, the study by Marret et al., shown that wash-out time and half wash-out time were dramatically greater in ovarian malignancies than in benign tumors. Thus, by now, there is no consensus quantitative parameters of CEUS for the discrimination of begin or malignant adnexal masses. In addition, there are technical limitations for acquisition of a clip for quantitative analysis, as any movement of the probe or the patients would impact on the results [19].

In the present study, the acquisition of imaging clip for qualitative analysis is very practical and less time-consuming, and the imaging information would not be disturbed by probe or body movement. Also, it is easier to manipulate the transabdominal probe to obtain more information, which might reduce subjective bias by using normal tissue as a reference standard and enhance operators’ confidence for making differential diagnosis. With the use of both the enhancement time and degree of the adnexal masses and the comparison with uterine myometrium, the enhancement time and degree, the agent distribution patterns and dynamic changes of enhancement can be assessed simultaneously, which is a different approach from previous published studies [19]–[21], [27]–[28]. Our current study found that malignant tumors were characterized by more rapid enhancement and a higher level of enhancement than benign lesions. Earlier or simultaneous enhancement compared with the myometrium was found in 93.8% (45/48) of malignant tumors and 12.5% (9/72) of benign masses. Hyper-or iso-enhancement was present in the most (97.9% 47/48) of malignant tumors. This phenomenon might be related to the high-velocity flow through the arteriovenous shunts that are typical malignant neovascularization and seems to be more certain in carcinomas, where the increased vascularization is thought to decrease the arrival time of the contrast agent and increase the enhancement intensity. These findings appear to be consistency with those previously reported by Sconfienza et al [20], Orden’ et al. [27] and Marret et al. [28] although in the Marret’s study the findings did not prove to be statistically significant. In addition, the inhomogeneous enhancement was observed in the most 91.7% (44/48) of malignant tumors, whereas only in 6.9% (5/72) of benign masses, which might be a key predictor for the discrimination between malignant and benign adnexal masses.

In our study, there were two contrast imaging features, i.e., earlier or simultaneous contrast arriving time and heterogeneous enhancement pattern found in most malignant masses. When adopting both of them as the diagnostic criterion for discrimination, the accuracy of the technique for the diagnosis of the malignant tumors and Youden’s index were 93.3% (112/120) and 0.88, respectively, achieving the highest diagnostic capability in comparison with other features or combinations. It should be point out that some inflammatory lesions could have similar enhancement features as malignant tumors. This feature might be related to the increased flow velocity caused by inflammatory factors.

There are some limitations of this study. First, some patients were excluded if the mass cannot be displayed in the same imaging plane for CEUS. Second, the imaging analysis was carried out in consensus fashion instead independently performed by readers, which might lead to bias even though this method is widely used in clinical settings. Further interobserver and intraobserver variability studies are needed to assess the reliability and acceptance of qualitative CEUS method. The comparison study between conventional US and CEUS in characterization was not performed in this study. Further study is needed.

In conclusion, the preliminary findings indicate that transabdominal CEUS is useful in the discrimination between malignant and benign adnexal diseases by using qualitative parameters of dynamic contrast imaging. Earlier or simultaneous enhancement with inhomogeneous pattern is key factors for highly suggestive of malignant masses.

## References

[1] Jeong YY, Outwater EK, Kang HK (2000) Imaging evaluation of ovarian masses, Ultrasound Obstet Gynecol. 20: 1445–1470. 10.1148/radiographics.20.5.g00se101445 10992033

[2] Valentin L, Ameye L, Jurkovic D, Metzger U, Lécuru F, et al (2006) Which extrauterine pelvic masses are difficult to correctly classify as benign or malignant on the basis of ultrasound findings, and is there a way of making a correct diagnosis? Ultrasound Obstet Gynecol 27: 438–444. 16526098 10.1002/uog.2707

[3] Timmerman D, Valentin L (2007) Imaging in gynecological disease. Ultrasound Obstet Gynecol 29: 483–484. 17444563 10.1002/uog.4025

[4] Yazbek J, Raju KS, Ben-Nagi J, Holland T, Hillaby K, et al (2007) Accuracy of ultrasound subjective pattern recognition for the diagnosis of borderline ovarian tumors. Ultrasound Obstet Gynecol 29: 489–495. 17444554 10.1002/uog.4002

[5] Twickler DM, Moscos E (2010) Ultrasound and assessment of ovarian cancer risk. Am J Radiol 2: 322–329. 10.2214/AJR.09.3562 20093591

[6] Togashi K (2003) Ovarian cancer: the clinical role of US, CT, and MRI. Eur Radiol 13: 87–104. 10.1007/s00330-003-1964-y 15018172

[7] Medeiros LR, Rosa DD, da Rosa MI, Bozzetti MC (2009) Accuracy of ultrasonography with color Doppler in ovarian tumor: a systematic quantitative review. Int J Gynecol Cancer 19: 230–236. 19395998 10.1111/IGC.0b013e31819c1369

[8] Kupesic S, Plavsic BM (2006) Early ovarian cancer: 3-D power Doppler. Abdom Imaging 31: 613–619. 16447081 10.1007/s00261-005-0398-1

[9] Guerriero S, Ajossa S, Piras S, Gerada M, Floris S, et al (2007) Three-dimensional quantification of tumor vascularity as a tertiary test after B-mode and power Doppler evaluation for detection of ovarian cancer. J Ultrasound Med 26: 1271–1278. 17901131 10.7863/jum.2007.26.10.1271

[10] Testa AC, Ajossa S, Ferrandina G, Fruscella E, Ludovisi M, et al (2005) Does quantitative analysis of three-dimensional power Doppler angiography have a role in the diagnosis of malignant pelvic solid tumors? A preliminary study. Ultrasound Obstet Gynecol 26: 67–72. 15971296 10.1002/uog.1937

[11] Jokubkiene L, Sladkevicius P, Valentin L (2007) Does three-dimensional power Doppler ultrasound help in discrimination between benign and malignant ovarian masses? Ultrasound Obstet Gynecol 29: 215–225. 17201017 10.1002/uog.3922

[12] Schneider M (1999) SonoVue, a new ultrasound contrast agent. Eur Radiol (Suppl 3): S347. 10.1007/pl00014071 10602926

[13] Phillips P, Gardner E (2004) Contrast-agent detection and quantification. Eur Radiol (Suppl 8): 4. 15700327

[14] Correas JM, Bridal L, Lesavre A, Méjean A, Claudon M, et al (2001) Ultrasound contrast agents: properties, principles of action, tolerance, and artifacts. Eur Radiol 11: 1316–1328. 11519538 10.1007/s003300100940

[15] Albrecht T, Blomley M, Bolondi L, Claudon M, Correas JM, et al (2004) Guidelines for the use of contrast agents in ultrasound. Ultrasound Med 25: 249–256. 10.1055/s-2004-813245 15300497

[16] Claudon M, Cosgrove D, Albrecht T, Bolondi L, Bosio M, et al (2008) Guidelines and good clinical practice recommendations for contrast enhanced ultrasound (CEUS) - update 2008. Ultraschall Med 29: 28–44. 18270887 10.1055/s-2007-963785

[17] Hohmann J1, Skrok J, Basilico R, Jennett M, Müller A, et al (2012) Characterisation of focal liver lesions with unenhanced and contrast enhanced low MI real time ultrasound: on-site unblinded versus off-site blinded reading. Eur J Radiol 81: 317–324. 22100374 10.1016/j.ejrad.2011.10.015

[18] Testa AC, Timmerman D, Exacoustos C, Fruscella E, Van Holsbeke C, et al (2007) The role of CnTI-SonoVue in the diagnosis of ovarian masses with papillary projections: a preliminary study. Ultrasound Obstet Gynecol 29: 512–516. 17444549 10.1002/uog.4013

[19] Testa AC, Timmerman D, Van Belle V, Fruscella E, Van Holsbeke C, et al (2009) Intravenous contrast ultrasound examination using contrast-tuned imaging (CnTI) and the contrast medium SonoVue for discrimination between benign and malignant pelvic masses with solid components. Ultrasound Obstet Gynecol 34: 699–710. 19924735 10.1002/uog.7464

[20] Sconfienza LM, Perrone N, Delnevo A, Lacelli F, Murolo C, et al (2010) Diagnostic value of contrast-enhanced ultrasonography in the characterization of ovarian tumors. J Ultrasound 1: 9–15. 10.1016/j.jus.2009.09.007 PMC3552646 23396092

[21] Fleischer AC, Lyshchik A, Andreotti RF, Hwang M, Jones HW 3rd, et al (2010) Advances in sonographic detection of ovarian cancer: depiction of tumor neovascularity with microbubbles. Am J Roentgenol 194: 343–348. 20093594 10.2214/AJR.09.3446

[22] Timmerman D, Valentin L, Bourne TH, Collins WP, Verrelst H, et al (2000) Terms, definitions and measurements to describe the sonographic features of adnexal tumors: a consensus opinion from the International Ovarian Tumor Analysis (IOTA)Group. Ultrasound Obstet Gynecol 16: 500–505. 11169340 10.1046/j.1469-0705.2000.00287.x

[23] Valentin L (1997) Gray scale sonography, subjective evaluation of the color Doppler image and measurement of blood fow velocity for distinguishing benign and malignant tumors of suspected adnexal origin. Eur J Obstet Gynecol Reprod Biol 72: 63–72. 9076424 10.1016/s0301-2115(96)02661-9

[24] Guerriero S, Alcazar JL, Coccia ME, Ajossa S, Scarselli G, et al (2002) Complex pelvic mass as a target of evaluation of vessel distribution by color Doppler for the diagnosis of adnexal malignancies: results of a multicenter European study. J Ultrasound Med 21: 1105–1111. 12369665 10.7863/jum.2002.21.10.1105

[25] Piscaglia F, Nolsøe C, Dietrich CF, Cosgrove DO, Gilja OH, et al (2012) The EFSUMB Guidelines and Recommendations on the Clinical Practice of Contrast Enhanced Ultrasound (CEUS): Update 2011 on non-hepatic applications. Ultraschall Med 33: 33–59. 21874631 10.1055/s-0031-1281676

[26] Claudon M, Dietrich CF, Choi BI, Cosgrove DO, Kudo M, et al (2013) Guidelines and good clinical practice recommendations for contrast enhanced ultrasound (CEUS) in the liver–update 2012: a WFUMB-EFSUMB initiative in cooperation with representatives of AFSUMB, AIUM, ASUM, FLAUS and ICUS. Ultraschall Med. 34: 11–29. 10.1055/s-0032-1325499 23129518

[27] Orden MR, Jurvelin JS, Kirkinen PP (2003) Kinetics of a US contrast agent in benign and malignant adnexal tumors. Radiology 226: 405–410. 12563133 10.1148/radiol.2262011450

[28] Marret H, Sauget S, Giraudeau B, Brewer M, Ranger-Moore J, et al. (2004) Contrast-enhanced sonography helps in discrimination of benign from malignant adnexal masses. J Ultrasound Med 23: 1629–1639, quiz 1641–1642. 10.7863/jum.2004.23.12.1629 15557306

